# Host‐plant associated genetic divergence of two *Diatraea* spp. (Lepidoptera: Crambidae) stemborers on novel crop plants

**DOI:** 10.1002/ece3.2541

**Published:** 2016-11-13

**Authors:** Andrea L. Joyce, Miguel Sermeno Chicas, Leopoldo Serrano Cervantes, Miguel Paniagua, Sonja J. Scheffer, M. Alma Solis

**Affiliations:** ^1^ University of California MercedMercedCAUSA; ^2^ AgronomyUniversity of El SalvadorSan SalvadorEl Salvador; ^3^ Systematic Entomology LabUSDA‐ARSBeltsvilleMDUSA; ^4^ Systematic EntomologyNational Museum of Natural HistoryUSDAWashingtonDCUSA

**Keywords:** adaptation, biological control, biotypes, host plant phenology, host plant volatiles, host‐associated differentiation, parasitism

## Abstract

*Diatraea lineolata* and *Diatraea saccharalis* (Lepidoptera: Crambidae) are moths with stemboring larvae that feed and develop on economically important grasses. This study investigated whether these moths have diverged from a native host plant, corn, onto introduced crop plants including sorghum, sugarcane, and rice. *Diatraea* larvae were collected from these four host plants throughout the year in El Salvador and were reared on artificial diet until moths or parasitoids emerged. Adult moths were subsequently identified to species. Amplified fragment length polymorphisms (AFLPs) and mitochondrial DNA cytochrome oxidase I (COI) were used to examine whether or not there was genetic divergence of *D. lineolata* or *D. saccharalis* populations on the four host plants. Percent parasitism was also determined for each moth on its host plants. *D. lineolata* was collected from corn in the rainy season and sorghum in the dry season. *D. saccharalis* was most abundant on sugarcane in the rainy season and sorghum in the dry season. The AFLP analysis found two genetically divergent populations of both *D. lineolata* and *D. saccharalis*. Both moths had high levels of parasitism on their dominant host plant in the rainy season, yet had low levels of parasitism on sorghum in the dry season. The presence of two genotypes of both *Diatraea* spp. on sorghum suggest that host‐associated differentiation is occurring on this novel introduced crop plant.

## 1 Introduction

Insects that colonize novel host plants can undergo divergent selection possibly leading to host‐associated differentiation (Abrahamson et al., 2003; Craig et al., 1993; Dres & Mallet, 2002; Medina, 2012; Via, 1999; Vialatte et al., 2005). Populations in distinct habitats, such as different host plants, may diverge genetically even if they remain in contact, a process called ecological speciation (Nosil, 2012; Schluter, 2001). Along with oviposition preference and performance characters, life‐history parameters such as generation time (e.g., voltinism), parthenogenesis, and variation in diapause contribute to the divergence of host plant‐associated populations of insects (Craig et al., 1993; Dickey & Medina, 2010; Diehl & Bush, 1984; Wood et al., 1999). Studies have demonstrated host‐associated differentiation (HAD) for insects on native plants (Abrahamson et al., 2003; Dickey & Medina, 2010; Scheffer & Hawthorne, 2007; Stireman, Nason, & Heard, 2005) as well as for those on novel host plants (Feder, Hunt, & Bush, 1993; Forbes et al., 2009). Natural enemies of herbivores such as parasitoids may undergo cascading or sequential genetic divergence as they follow herbivores onto novel host plants (Stireman et al., 2005; Forbes et al., 2009), or herbivores may escape parasitism on their newly colonized host plants. Formation of host plant‐associated insect populations has been suggested to be more likely with longer associations between insects and novel host plants (Siemann, Rogers, & Dewalt, 2006). However, rapid adaptation and formation of host‐associated insect populations have occurred on introduced cultivated crop plants (Dres & Mallet, 2002; Vialatte et al., 2005; Feder & Forbes, 2010). Additional examples of HAD on novel plants are likely to be uncovered given the large number of plants and insects, which have been introduced outside of their native range.

Host plant‐associated differentiation of insect populations has been demonstrated in a number of systems. Native insects colonizing introduced crop plants have diverged in the course of two hundred years or so (Feder et al., 1993; Vialatte et al., 2005; Feder & Forbes, 2010). *Rhagoletis pomonella* (Walsh) (Diptera: Tephritidae) has shifted from the native plant hawthorne onto apple, an introduced crop transplanted from the Old World into the United States. Differences in phenologies between these two host plants contribute to isolating the host races of this insect (Feder & Forbes, 2010). The aphid *Sitobion avenae* Fabricius (Homoptera: Aphididae) has divergent genotypes on cultivated and uncultivated hosts (Vialatte et al., 2005), formed presumably in the last 100 years since its introduction into the United States. In Europe, corn was introduced in the last 500 years. Currently, there are two host plant races of *Ostrinia nubilalis* Hübner (Lepidoptera: Crambidae), the European corn borer, which have unique pheromone blends and oviposition preference for their natal hosts (Bethenod et al., 2005). Similarly, the fall armyworm *Spodoptera frugiperda* J.E. Smith (Lepidoptera: Noctuidae) has been found to have two host plant strains, one feeding on corn and one on the introduced host rice (Pashley, Hardy, Hammond, & Mihm, 1990). The moths mate at different times of night and have unique pheromone blends, which contribute to ecological isolation (Groot, Marr, Heckel, & Schofl, 2010).

The sugarcane borer moth, *Diatraea saccharalis* (Fabricius) (Lepidoptera: Crambidae), is native in the Western Hemisphere and broadly distributed especially in regions associated with sugarcane (Bleszynski, 1969; Box, 1931; CAB 1989; Dyar & Heinrich, 1927). Larvae are endophagous, developing in host plant stems until they pupate and emerge as adults. This moth is considered an introduced species in the southern United States and can be a pest of cultivated plants including corn (*Zea mays* L.), sorghum (*Sorghum bicolor* L.), and rice (*Oryza sativa* L.) in various parts of its range (Cherry & Nuessly, 1993; Fuchs, Huffman, & Smith, 1979; Gifford & Mann, 1967; Vargas, Lastra, & Solis, 2013; White et al., 2001). Few population genetic studies of this insect have been conducted (Joyce et al., 2014; Lange, Scott, Graham, Sallam, & Allsopp, 2004; Pashley et al., 1990). Pashley et al. (1990) found that populations of *D. saccharalis* from the southern United States and Mexico were divergent from those of Brazil. *D. saccharalis* has been considered to be a single species with a wide distribution, but a recent study suggests that at least three or more species may exist (Joyce et al., 2014). In the southern United States, at least two putative species were found to occur, one in Texas and Louisiana, and a divergent genotype in Florida (Joyce et al., 2014). Exploring the genetic variability of *D. saccharalis* in a potential region of origin such as Central America would permit investigation of whether divergence has occurred between populations occurring on native crops such as corn or introduced crops. In Central America, another *Diatraea* species, *D. lineolata* (Walker) (Lepidoptera: Crambidae) also feeds on corn (Box, 1951; Quezada, 1978; Solis, 2004; Solis & Metz, 2015), a crop plant considered native in Mexico and Central America and domesticated in the last 5,000–10,000 years (Matsuoka et al., 2002). Because both *D. saccharalis* and *D. lineolata* are associated with corn and also feed on introduced crops, they may be subject to disruptive selection from crop plants introduced in the last few hundred years.

El Salvador has a tropical climate with a pronounced dry season and is classified as tropical savannah (Peel, Finlayson, & McMahon, 2007). During the rainy season from May until October, about 95% of annual rainfall occurs, with the months of April and November serving as transition months to and from the dry season. A widespread cropping rotation pattern in El Salvador is corn cultivation in the rainy season, followed by sorghum (an introduced crop) in the dry season (Quezada, 1978; Serrano‐Cervantes et al., 1986). Rice and sugarcane, both nonnative cultivars, are also planted in the rainy season, while plantings of sugarcane persist throughout the year during all 12 months, essentially being cultivated as a perennial crop plant. As the tropical climate transitions from the rainy to the dry season and plants dry out, developing larvae of both moth species become quiescent and shift to a dormant form where they no longer feed and larvae lose their pigmented colored spots (pinacula) on the cuticle (Dyar & Heinrich, 1927; Kevan, 1944; Quezada, 1978). The climatic extremes between the rainy and dry season along with novel host plant availability could exert strong selective pressure on insect populations, contributing to insect populations adapting to novel host plants.

The objective was to determine whether either of two moth species, *D. saccharalis* and *D. lineolata*, has genetically divergent host plant‐associated strains feeding on a native host, corn, and the introduced Old World crop plants, sugarcane, rice or sorghum. To test this, *D. lineolata* and *D. saccharalis* larvae were collected from available host plants in the rainy season and the dry season in El Salvador, Central America. If host‐associated differentiation had occurred on novel host plants, insects from two host plants (such as corn and sorghum) in the same location should be more genetically divergent than insects collected from a single host plant species such as corn from distant geographic locations.

## 2 Materials and Methods

### 2.1 Geographic location of study and field collection of larvae from host plants

The study was conducted in El Salvador, Central America, from August 2011 to May 2012 with an additional collection in rice in October 2013. We only used adult moths reared from field‐collected, host plant‐associated larvae. At each field site, host plants were searched for evidence of larval feeding in stems, indicated by insect frass exuding from a hole in the plant stem. Host plant stems with larvae were cut, and larvae were removed and placed individually in 60‐ml plastic cups on artificial diet (Southland Products, Lake Village Arkansas) in order to rear larvae to adult moths. Larvae were transported to the laboratory for rearing at the University of El Salvador and observed at least twice a week. Any adult moths or parasitoid wasps or flies that emerged from larvae were preserved by freezing or by storage in 80% ethanol. We used the emerged parasitoids to determine the parasitism rate for each *Diatraea* species on each plant type by dividing the number of parasitoids emerged by the sum of moths and parasitoids emerged.

### 2.2 Identification of adult moths by morphology

Reared adult *Diatraea* were identified to species by examining the adult male and female genitalia and comparing them to the key by Dyar and Heinrich (1927). Abdomens of adult moths were prepared for study by soaking the abdomen in cold 10% potassium hydroxide (KOH) overnight to be able to study the sclerotized structures for identification of the genitalia (Robinson, 1976). They were then placed in polyethylene genitalia vials with glycerin for future study and slide mounting. Voucher specimens are at the National Museum of Natural History, Smithsonian Institution, Washington, DC.

### 2.3 DNA extraction

Following the identification of adult moths, the six legs of each moth were used for DNA extractions using the Qiagen DNeasy Blood and Tissue kit (Venlo, Netherlands) following the protocols for animal tissue with an incubation time of 2 h at 65°C (Qiagen 2006). Final products were eluted in 100 μl of AE buffer. The DNA quantity was measured using the Qubit^®^ dsDNA HS Assay kit (Life Technologies). The quantity of DNA in samples averaged 2–5 ng of DNA per μl. Both male and female adults were used for molecular work.

### 2.4 Population genetics: amplified fragment length polymorphisms (AFLPS)

Amplified fragment length polymorphisms were produced as described by Vos et al. (1995) and Joyce et al. (2010). Two primer combinations were used, (1) M‐CAT and E‐ACT, and (2) M‐CAA and E‐ACT. Individuals from the four host plant populations (corn, sugarcane, sorghum, and rice) were randomized on two 96‐well plates for AFLP reactions.

Each restriction ⁄ ligation reaction (well) consisted of the following: 0.05 μl each of EcoRI and MseI, 1.1 μl of T4 DNA ligase buffer, 1.1 μl of 0.5 mol/L NaCl, 0.55 μl of diluted BSA (bovine serum albumin), 0.03 μl of T4 DNA ligase, 1.0 μl each of EcoRI and MseI adaptor pairs (Life Technologies‐Thermo Fisher Scientific, Waltham, MA, USA), and 0.61 μl of sterile distilled water. The plate with restriction⁄ ligation reactions was held at room temperature overnight (ca. 12 h at 25°C) to ensure complete digestion (Saunders, Mischke, & Hemeida, 2001). The amplified product was diluted 20‐fold using 15 mmol/L Tris‐HCl buffer (pH 8.0) containing 0.1 mmol/L EDTA. Pre‐selective PCR amplification was performed on a ThermoFisher Arktik thermal cycler. Each reaction contained 15 μl of AFLP preselective mix (all Life Technologies/Thermo Fisher), 1 μl of each amplification primer (Life Technologies), along with 4 μl of the diluted restriction⁄ ligation mixture. The PCR program for pre‐selective amplification consisted of an initial warm‐up of 95°C for 1 min followed by 20 cycles at 95°C for 20 s, 56°C for 30 s, and 72°C for 90 s with a final hold at 75°C for 5 min. The amplified product was diluted 20‐fold using 15 mmol/L Tris‐HCl buffer (pH 8.0) containing 0.1 mmol/L EDTA. Selective amplification was conducted using two primer combinations. For each selective amplification, a reaction consisted of 15 μl of AFLP platinum supreme mix, 1.0 μl of EcoRI + 3 selective primer, and 1.0 μl of MseI + 3 selective primer (all Life Technologies). The PCR program for selective amplification consisted of an initial warm‐up of 95°C for 1 min, 12 cycles of 95°C for 20 s, 65°C for 40 s with a lowering of 0.7°C per cycle, 72°C for 90 s, followed by 35 cycles of 95°C for 20 s, 56°C for 40 s, 72°C for 90 s, and finally a hold of 72°C for 7 min before storing the samples at 4°C. Prior to capillary electrophoresis, 0.4 μl of the GeneScan LIZ 500 size standard and 0.9 μl of HiDi formamide (all Life Technologies) were added to 1 μl of the final product of each sample. Sample fragments were separated using automated capillary electrophoresis by the ABI 3730 XL automated capillary DNA sequencer. GeneMapper version 5.0 (Life Technologies) was used to determine presence or absence of fragments. The peak detection threshold was set for each primer combination and was typically 100 luminescent units. Each AFLP marker was considered a locus and assumed to have two possible alleles (0 = absent, 1 = present). Bands not present in more than one individual were eliminated (i.e., private alleles) prior to further analyses, as they were not considered informative. Structure 2.3.4 software (Pritchard, Wen, & Falush, 2007) was used to group individuals with similar genotypes within each species. Structure uses a Bayesian algorithm to cluster individuals into *K*, which is defined as the number of genetically distinct populations in a data set. Parameters used for the analyses include the following: no a priori assignment of individuals to a known population, analysis for diploid insects, a burn‐in of 10,000 iterations, an admixture model, and independent loci.

**Figure 1:**
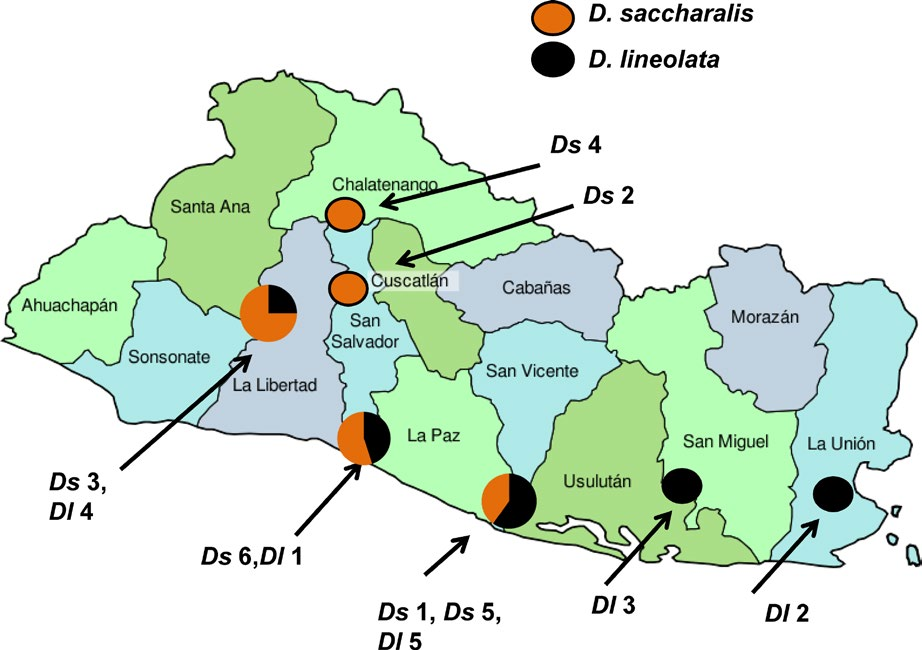
Collection site codes correspond to the collection locations on Tables 1 and 2 for each *Diatraea* species in El Salvador. See Tables 1 and 2 for collection details including date, location name, longitude, latitude, and host plant type. The proportion of *Diatraea saccharalis* and *Diatraea lineolata* collected at each site are shown in brown (*Ds*) or black (*Dl)*

If collection locations were fewer than 5 miles apart, they were considered one location for the population genetic analysis of AFLPs with Structure software (Figure 1). The following two sites were combined and considered one collection site for genetic analysis with Structure; Cooperative El Nilo, and Santa Cruz Porrillo (*D. saccharalis* sites 1, 5). Five collecting locations were used for Structure genetic analysis for both *D. lineolata* and *D. saccharalis* (Figure 1). The number of potential populations for *K* was estimated as the number of geographic sampling locations (5) plus 4 (*K* = 9) for both species as suggested by Pritchard, Stephens, and Donnelly (2000), and each iteration was run 20 times. At the completion of Structure runs, *K* was calculated for each species using Structure Harvester (Earl & VonHoldt, 2012; Evanno, Regnaut, & Goudet, 2005), to determine the most likely number of population clusters (*K*) for each species.

Analyses of molecular variance (AMOVA) tests were run using the AFLP data using GenAlEx 6.0 (Peakall & Smouse, 2006). For *D. lineolata*, all individuals collected were included in a comparison of host plant‐associated individuals collected from corn and sorghum (Table 1). The individuals collected from corn were obtained in the rainy season, and those from sorghum were collected in the dry season; the AMOVA of season consists of the same individuals and produced identical results as that obtained when comparing host plant populations. A separate AMOVA was run to examine genetic variation among four host plant–site populations. We did not include sites with less than five individuals in AMOVA; therefore, the one adult *D. lineolata* from site 3 in San Miguel was not included. The four host plant–site populations compared were (1) corn at UES‐EE, (2) corn at La Union, (3) sorghum at La Union, and (4) sorghum at Santa Cruz Porillo. The host plant–site analysis was chosen as there was an unbalanced design with different numbers of host plants at each site (Sword, Joern, & Senior, 2005). Pairwise comparisons were made of the genetic distance (*F*_ST_) and significance among the four host plant–site populations. All AMOVAs were run with 999 permutations. For *D. saccharalis*, AMOVA was run to compare three host plant‐associated populations, rice, sugarcane, and sorghum (corn was not included as there were only 3 *Ds* adults). Subsequently, four host plant–site populations were compared; (1) rice at El Nilo, (2) sugarcane at Ingenio La Cabana El Paisnal, (3) sorghum at San Andres Centa, and (4) sorghum at Santo Tomas. Similarly, (*F*_ST_) values were determined for pairs of populations and their significance was tested. An AMOVA was also run to compare variation of *D. saccharalis* populations between the rainy season and the dry season. Finally, for each *Diatraea* species, a Mantel test was run for the four host plant–site populations to determine whether genetic distance was significantly correlated with geographic distance.

**Table 1:** Collection sites for *Diatraea lineolata* larvae in El Salvador by host plant

Site number	Site code	Collection date 2011–2012	Identification by morphology	Host plant	Collection site	Municipio, departmento	Latitude/Longitude
1	UES‐corn	Sept. 8, 2011	*D. lineolata*	Corn	UES, Estacion Experimental	San Luis Talpa, La Paz	N13°28′, W89°06′
		Oct. 7, 2011	*D. lineolata*	Corn	UES, Estacion Experimental	San Luis Talpa, La Paz	N13°28′, W89°06′
2	LaU‐corn	Oct. 12, 2011	*D. lineolata*	Corn	Canton Sirama	Sirama, La Union	N13°21′, W87°54′3″
		Jan. 11, 2012	*D. lineolata*	Sorghum	Canton Sirama	Sirama, La Union	N13°21′, W87°54′3″
3	SM‐sorg	Sept. 29, 2011	*D. lineolata*	Sorghum	Lotte Amatillo	San Miguel	N13°20′29.554, W88°16′4.957
4	SaC‐sorg	Dec. 19, 2011	*D. lineolata*	Sorghum	CENTA Field 1, Lot 11	San Andes La Libertad	N13°48.365′, W89°23.71
5	SCP‐sorg	Feb. 7, 2012	*D. lineolata*	Sorghum	CENTA Research Station	Santa Cruz Porrillo, San Vicente	N13°26′12.2, W88°48.10.7
		March 7, 2012	*D. lineolata*	Sorghum	CENTA Research Station	Santa Cruz Porrillo, San Vicente	N13°26′12.2, W88°48.10.7

UES, University of El Salvador; CENTA, Centro Nacional de Tecnologίa Agropecuaria y Forestal.

### 2.5 Mitochondrial DNA‐COI

A 658‐base pair region (the “bar code”) of the mitochondrial COI gene region was sequenced from 26 *D. lineolata* and 23 *D. saccharalis* including a few individuals from each collection site (Tables 1 and 2, Figure 1). The DNA used for sequencing COI was extracted as described above. The bar‐code region of the COI gene was amplified using primers for the mitochondrial DNA “bar code” of Lepidoptera (Hajibabaei, Janzen, Burns, Hallwachs, & Hebert, 2006). The forward primer LepF was 5_‐ATTCAACCAATCATAAAGATATTGG‐3, and the reverse primer sequence of LepR was 5_‐ TAAACTTCTGGATGTCCAAAAAATCA‐3 (Hajibabaei et al., 2006). The touchdown PCR program consisted of an initial 2 min at 95°C, then 12 cycles of 95°C for 10 s, 58–46°C for 10 s with a lowering of 1°C temperature each cycle, and 72°C for 60 s. Following PCR and confirmation of amplification on an agarose gel, samples were cleaned up using a USB Exo‐sapit pcr cleanup kit (Affymetrix, Inc., Santa Clara, Cal.). Sequencing was carried out on an ABI 3730XL Genetic Analyzer. DNA sequences were edited using Geneious 7.0 (Biomatters, Aukland, New Zealand) (Kearse et al., 2012). The forward and reverse sequences for each individual were trimmed and assembled into a consensus sequence. Mitochondrial DNA was compared with the COI sequences available for *D. saccharalis* available in GenBank and the Bar Code of Life Data System (BOLD) (Milton, Pierossi, & Ratnasignham, 2013). We did not find any existing *D. lineolata* mitochondrial DNA sequences in GenBank or BOLD available for comparison. We aligned our 23 consensus sequences from *D. saccharalis* with others previously obtained from *D. saccharalis* from the southern USA (Joyce et al., 2014) and with 34 other *D. saccharalis* COI sequences available in GenBank and BOLD. Alignments were made in Geneious 7.0 using the Clustal W alignment function and used to make an unrooted neighbor‐joining tree. Bootstrap support values were obtained by 500 pseudoreplicates of the aligned dataset.

**Table 2:** Collection sites for *Diatraea saccharalis* larvae by host plant

Site Number	Site code	Collection date 2011–2012, 2013	Identification by morphology	Host plant	Collection site	Municipio, department	Latitude/Longitude
1	Nil‐rice	Oct. 7, 2011	*D. saccharalis*	Rice	El Nilo 1	Zacatecaluca, La Paz	N13°23.700, W 88°52.860
		Oct. 18, 2013	*D. saccharalis*	Rice	El Nilo 1	Zacatecaluca, La Paz	N13°23.700, W88°52.860
		Dec. 12, 2011	*D. saccharalis*	Cane	El Nilo 1	Zacatecaluca, La Paz	N13°23.700′, W88°52.860
		Feb. 12, 2012	*D. saccharalis*	Sorghum	El Nilo 1	Zacatecaluca, La Paz	N13°23.700′, W88°52.860
2	InC‐cane	Nov. 22, 2011	*D. saccharalis*	Cane	Ingenio La Cabaña	Paisnal, San Salvador	N13°59′56.8″, W 89°11′09.5″
		Dec. 6, 2011	*D. saccharalis*	Cane	Ingenio La Cabaña	Paisnal, San Salvador	N13°59′56.8″ W89°11′09.5″
3	SaC‐sorg	Dec. 19, 2011	*D. saccharalis*	Sorghum	CENTA	San Andres, La Libertad	N13°48.365′, W89°23.717′
4	CJC‐sorg	Jan. 26, 2012	*D. saccharalis*	Sorghum	Cooperative Juan Chacon	Chilamate, Chaletanango	N14°5.310′, W89°12.582
5	SCP‐sorg	Feb. 7, 2012	*D. saccharalis*	Sorghum	CENTA	Santa Cruz Porrillo, San Vicente	N13°26′12.2, W88°48.10.7
		March 7, 2012	*D. saccharalis*	Sorghum	CENTA	SCP, SV	N13°26′12.2, W88°48.10.7
6	SaT	March 28, 2012	*D. saccharalis*	Sorghum	Santo Tomas	Santo Tomas, La Paz	N13°28′35.23″ W89°6′33.32″
		April 19, 2012	*D. saccharalis*	Corn	Santo Tomas	St. Tm, La Paz	N13°28′35.23″, W89°6′33.32″

CENTA, Centro Nacional de Tecnologίa Agropecuaria y Forestal.

## 3 Results

*Diatraea lineolata* and *D. saccharalis* larvae were collected at sites throughout El Salvador (Tables 1 and 2, Figure 1). We attempted to visit collection sites multiple times so that we could collect from host plants cultivated throughout the year in the rainy season and the dry season. *Diatraea lineolata* larvae collected from the rainy season had black pinacula (spots), while larvae of *D. saccharalis* from the rainy season had brown pinacula. Larvae of both species collected in the dry season typically had pale cream colored pinacula that blended in color with the integument. All larvae were reared as previously described, and subsequent identification of adults (described below) found that larvae with black pinacula were *D. lineolata*, while larvae with brown pinacula were *D. saccharalis*.

**Figure 2:**
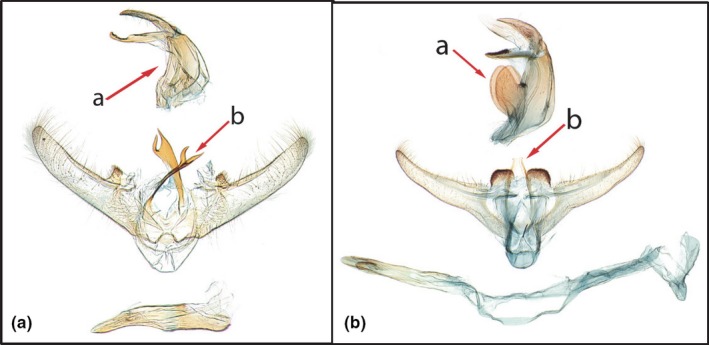
(a) *Diatraea lineolata* male genitalia, USNM #113649, (a.a) lateral lobe of tegumen absent; (a.b) apex of juxta arms with two distinct points, apical teeth subequal in size, and clawlike (b) *Diatraea saccharalis* male genitalia, USNM #114649, (b.a) lateral lobe of tegumen present, round, and as long as wide; (b.b) apex of juxta arms blunt, not bidentate

Adult identification was confirmed using genitalia and the key by Dyar and Heinrich (1927) (Figure 2a,b). *Diatraea lineolata* was collected from corn in the rainy season, and sorghum in the dry season, but was not found in any collections from sugarcane or rice. *Diatraea saccharalis* was collected from sugarcane and rice in the rainy season, and sorghum and corn in the dry season. Although *D. saccharalis* was collected in rice, another stemborer *Rupela albinela* (Cramer) (Lepidoptera: Pyralidae) was much more abundant in rice.

### 3.1 Parasitism of *Diatraea lineolata* and *Diatraea saccharalis*

Parasitoid wasps and parasitoid flies were reared from the collected *Diatraea* larvae. *D. lineolata* were collected from corn and sorghum, yet 37% parasitism of *D. lineolata* occurred on corn, with only 7% parasitism on sorghum (Table 3). Two parasitoid flies, *Billea* (*Paratheresia* spp.) and *Palpozenillia* (Diptera: Tachinidae), were the dominant parasitoids of *D. lineolata* on corn. *Diatraea saccharalis* was parasitized most heavily on sugarcane with 67% parasitism, with most parasitism by *Billea* spp. (Table 3). Only 5% parasitism of *D. saccharalis* occurred on sorghum and 10% on rice (Table 3).

**Table 3:** Number of adult *Diatraea lineolata* and *Diatraea saccharalis* moths or parasitoids reared from larvae on the four host plants

Moth species	Parasitoid	Host plant			
		Corn	Sugarcane	Sorghum	Rice
*D. lineolata*		22	–	43	
	*Billea* spp.	5	–	3	–
	*Palpozenillia* spp.	7	–	0	–
	Hyperparasitoid	1	–	0	–
Total percent parasitism	(Par/Moth + Par)	37%	–	7%	–
*D. saccharalis*		3	20	19	10
	*Billea* spp.	0	32	0	0
	*Apanteles* spp.	0	1	0	0
	*Iphaulaux* spp	0	1	0	0
	Hyperparasitoid	0	7	0	0
	Tachinidae	0	0	1	0
	Ceraphronidae	0	0	0	1
Total percent parasitism	(Par/Moth + Par)	0%	67%	5%	10%

*Billea, Palpozenillia* = (Diptera: Tachinidae), Others = Hymenoptera. No *D. lineolata* were found in collections from sugarcane or rice.

### 3.2 Population comparisons using AFLPs

**Figure 3:**
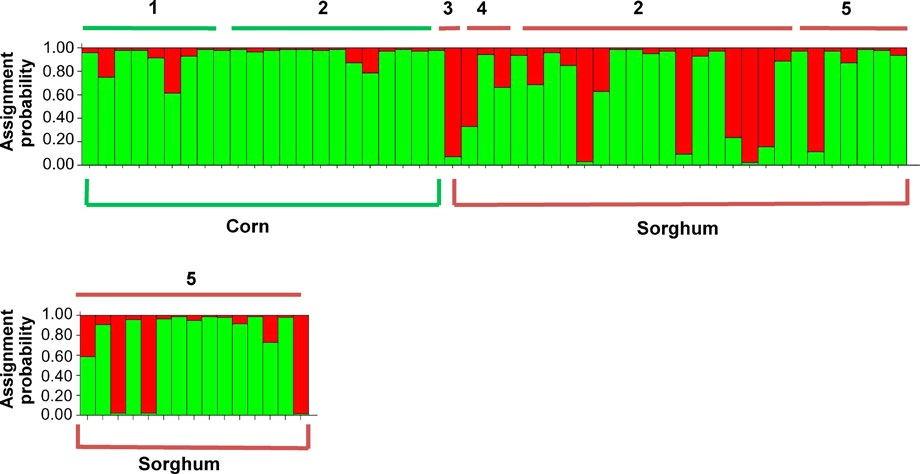
Structure analysis of AFLPs from *Diatraea lineolata* collected in El Salvador from corn and sorghum from September 2011 to March 2012. Structure 2.3.4 was run using the following parameters: diploid individuals, 10,000 iterations, admixed data, and independent loci. Structure Harvester found that *K* = 2. The number above the bars represents the collection site for larval collections (see Table 1, Figure 1). The host plant that each larva was collected from is below the bars

**Figure 4:**
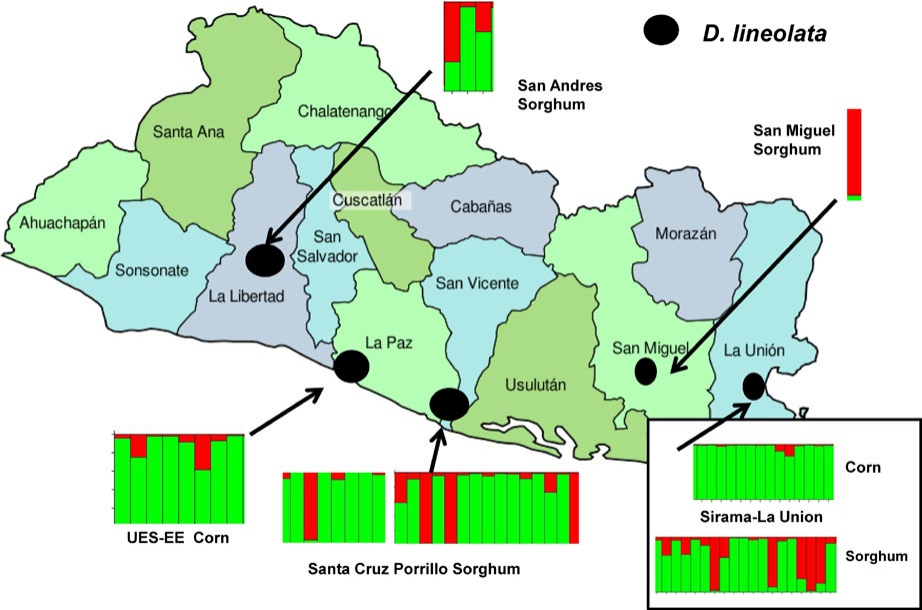
Map of El Salvador with results from Structure analysis for individual *Diatraea lineolata* moths collected on corn or sorghum, mapped to their respective collection sites

We produced 125 unique AFLP markers using two primer combinations for 65 *D. lineolata* moths. The 65 individuals consisted of 22 adults reared from corn and 43 adults reared from sorghum. Individuals from corn were reared from two sites (Table 1), while individuals from sorghum were obtained from three sites (Table 1). Structure Harvester found that *K* = 2, indicating two genetically distinct groups of *D. lineolata* (Figure 3). Collections from corn were assigned primarily to one cluster (green, Figure 3), while collections from sorghum had two genetically distinct clusters (red and green, Figure 3). To visualize geographic variation in genotypes, the individual bars from structure, which represent individual moths, were mapped to their collection sites (Figure 4). Individuals reared from corn came from the University of El Salvador Experimental Station in San Luis Talpa and also from Canton Sirama in La Union. *D. lineolata* collected from corn from geographically distant sites are almost entirely assigned to the same genetic cluster illustrated in green. At Canton Sirama in La Union, individuals collected from corn and sorghum had more genetic differentiation (with no geographic separation) than those collected from corn at two sites at a substantial distance from each other (Figure 4, *see AMOVA results below*).

**Figure 5:**
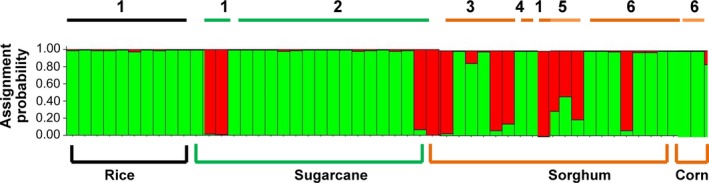
Structure analysis for *Diatraea saccharalis* moths raised from larvae collected from host plants throughout the year. Each vertical bar represents an individual. Collection site numbers are above the bars, while host plants are below the bars. Details on collections are listed in Table 2. Structure Harvester found that *K* = 2

For the second moth species, *D. saccharalis*, we produced 112 AFLP markers for 52 adults reared from larvae collected on rice, sugarcane, sorghum, and corn. For the Structure software analysis, we used 10 adults reared from larvae collected on rice, 20 adults from sugarcane, 19 adults from sorghum, and three from corn. In the rainy season, sugarcane was the dominant host plant of *D. saccharalis*, and *D. saccharalis* was not found on corn in the rainy season as we had expected. Collections from corn were dominated by the other *Diatraea* species, *D. lineolata*. Structure Harvester found for *D. saccharalis* that *K* = 2, indicating there were two genetically distinct groups (Figure 5). Samples from rice from two sites were all assigned to the green cluster, as were those from corn. Most individuals reared from sugarcane in the rainy season were assigned to the green cluster; collections from sorghum had a mix of two genotypes (Figure 5).

### 3.3 Analysis of molecular variation (AMOVA)

AMOVA of host plant‐associated populations of *D. lineolata* found that host plants had a significant effect on genetic variation (Table 4); host plant populations accounted for 2% of variation, while host plant × site populations contributed 6% of variation. Pairwise *F*_ST_ values between the four groups found that genetic distance between the two corn populations (UES, La Union, 0.024) was not significant, but genetic distances between corn and sorghum populations were significant (0.039–0.082), with the *F*_ST_ genetic distance of 0.068 between the corn and sorghum populations at the Canton Sirama (Table 5). A Mantel test found no significant relationship between the genetic distance and geographic distance for *D. lineolata* populations from corn and sorghum (*r* = .201, *p *= .501).

**Table 4:** Analysis of molecular variation (AMOVA) for *Diatraea lineolata* and *Diatraea saccharalis*

	Population	*df*	SS	Variation (%)	*p*
*Diatraea lineolata*	(a) Among host plant × site	3	77.468	6	.001
	Individuals within groups	57	786.630	94	
	(b) Among host plants	1	23.78	2	.007
	Individuals within groups	59	840.810	98	
*Diatraea saccharalis*	(a) Among host plant × site	3	80.794	8	.001
	Individuals within groups	40	543.933	92	
	(b) Among host plants	2	44.946	4	.001
	Individuals within groups	46	638.850	96	
	(c) Among seasons	1	29.557	4	.003
	Individuals within seasons	47	654.239	96	

**Table 5:** Pairwise comparisons of genetic divergence estimates (*F*_ST_) between *Diatraea* host plant–site populations

	Host plant × Site	1	2	3	4
*D. lineolata*	1. UESEE–Corn	0			
	2. La Union–Corn	0.024 NS	0		
	3. La Union–Sorghum	0.082^a^	0.068^a^	0	
	4. Santa Cruz Porrillo–Sorghum	0.039^a^	0.082^a^	0.035^a^	0
*D. saccharalis*	1. Nilo–Rice	0			
	2. Paisnal–Sugarcane	0.081^a^	0		
	3. San Andres–Sorghum	0.072^a^	0.104^a^	0	
	4. Santo Tomas–Sorghum	0.025NS	0.133^a^	0.095^a^	0

a *p* < .05. Comparison between populations is significant.

For *D. saccharalis*, the AMOVA test found the three host plant‐associated populations on rice, sugarcane and sorghum accounted for 4% of the variation, and season of collection (rainy or dry season) similarly accounted for 4% of variation. The host plant × site populations accounted for 8% of variation (Table 4). All AMOVA were significant (*p* < .01) (Table 4). The pairwise comparisons of *F*_ST_ values between the three host plant‐associated populations found that sugarcane population was significantly divergent from sorghum and marginally divergent from rice (sugarcane vs. sorghum *F*_ST_ 0.044, *p* = .01; rice vs. sugarcane *F*_ST_ 0.046, *p* = .05; rice vs. sorghum *F*_ST_ 0.018, *p* = .16). Pairwise comparisons of *F*_ST_ values for the four host plant–site populations found *D. saccharalis* on sugarcane was significantly divergent from the rice populations (*F*_ST_ = 0.081) and the two sorghum populations (*F*_ST_ 0.104, *F*_ST_ 0.133) (Table 5). Genetic divergence was significant between rice and sorghum from San Andres, but not significant between the rice population and sorghum from Santo Tomas; finally, there was significant divergence between sorghum from San Andres and sorghum from Santo Tomas (*F*_ST_ 0.095). A Mantel test found there was no significant correlation of genetic and geographic distance (*r* = .505, *p* = .105).

### 3.4 Mitochondrial DNA‐COI sequences

We obtained mitochondrial COI sequences from 26 individuals of *D. lineolata* including a few from each collection site. There was little genetic variation between pairs of sequences of mitochondrial DNA. Most individuals of *D. lineolata* were 99% or more similar, with 1–2 base pairs differing among some pairs. No previously sequenced COI sequences were available in GenBank for *D*. *lineolata* for comparison with our results.

**Figure 6:**
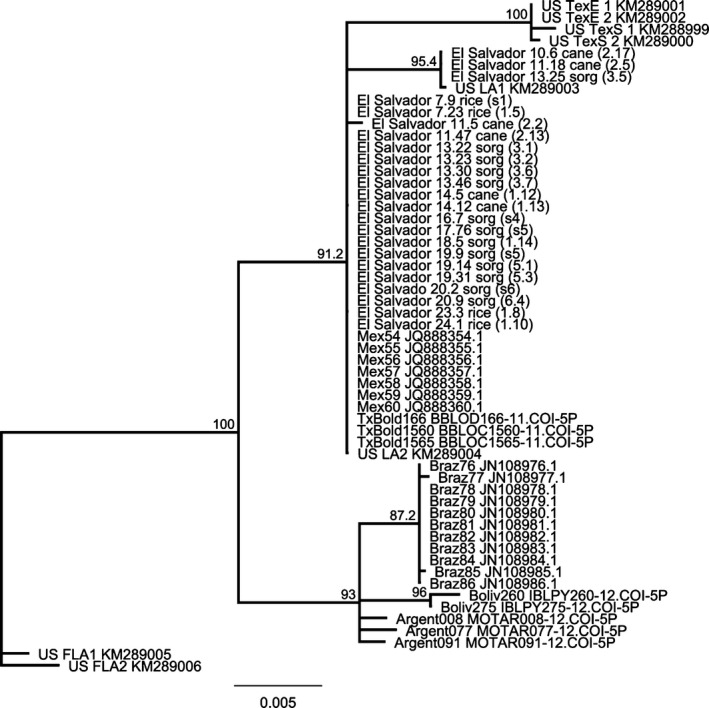
*Diatraea saccharalis* collected in El Salvador combined with individuals previously sequenced from GenBank and Bold. Bootstrap support values are based on 500 pseudoreplicates, and those above 80% are shown above supported nodes. Previously sequenced individuals have GenBank accession numbers or Barcode of Life identification numbers. El Salvador individuals are followed by an original collection number, followed by host plant of collection, and then collection site. Collection sites correspond to the six sites listed in Table 2. Collection sites followed by a number (i.e., rice 1.5) correspond to the individual bars in the AFLP structure analysis in Figure 5

For *D. saccharalis*, 23 individuals were sequenced including at least one individual from each collection site (Figure 6). Phylogenetic analysis of *D. saccharalis* sequences, including 34 sequences from GenBank, uncovered substantial geographic structure across the species (Figure 6). The unrooted neighbor‐joining consensus tree had three clades. The first and largest group of *D. saccharalis* consisted of individuals from El Salvador clustered with those from Mexico and the southern United States. Individuals from El Salvador in the larger clade were collected from all sites in the study and were collected on rice, sugarcane, and sorghum. Collections from El Salvador had a small subgroup of several individuals with 4–5 base pairs differing between them. These divergent individuals came from two interior collections (sites 2, 3) from sugarcane and sorghum. The 4‐to 5‐base pair difference in the sequences from the smaller clade represents almost a 1% divergence from other individuals collected in El Salvador (Table S1). An additional small group of four individuals from East and South Texas were also 1% divergent from other *D. saccharalis* in that clade. The second large grouping in the phylogenetic tree consists of individuals from South America, including Brazil, Argentina, and Bolivia. This group of moths was 2–3% divergent from the other two clades (Table S1); similarly, the third group of *D. saccharalis* from Florida was 2–3% divergent from the other two clusters.

## 4 Discussion

*Diatraea* larvae were collected from host plants and reared to adults to provide definitive evidence of their host plant association. *D. lineolata* was the predominant *Diatraea* species feeding on corn. *Diatraea saccharalis* was common on sugarcane in the rainy season and occasionally found on rice. Both *D. saccharalis* and *D. lineolata* were abundant on sorghum in the dry season, with nearly twice as many larvae of *D. lineolata* collected from sorghum as on were collected on corn. *Diatraea lineolata* is native to the Western Hemisphere and has had a much longer association with corn than with sorghum, which was introduced from Africa in the mid‐1800s (Dillon et al., 2007). The association of *Diatraea* spp. and sorghum in Central America is relatively recent on an evolutionary time scale, and little genetic variability would be expected in the mitochondrial DNA between individuals of *D. lineolata* collected on corn or sorghum. Most mitochondrial DNA sequences obtained from the *D. lineolata* reared in this study were nearly identical. In contrast, results from the AFLP markers for *D. lineolata* from corn and sorghum found the presence of two genetically distinct groups (Figure 3), which was not related to geographic distance among the populations. Individuals collected on corn represented one genetic group, while those collected from sorghum had two genetic groups present. The AFLP data suggest the populations of *D. lineolata* may be experiencing some degree of host plant‐associated differentiation.

Two populations of *D. lineolata* from La Union from corn and sorghum were collected several months apart in the same field, yet the sorghum field had an additional genotype present which was not collected in corn fields (Figure 3). The corn and sorghum populations from La Union had an *F*_ST_ value of 0.082, higher than the genetic distance between other host‐associated populations of *D. lineolata*. *D. lineolata* populations on corn are synchronized with the host plant, which is typically grown in May through November. As plants senesce, *D. lineolata* pupae enter diapause in plant stems or roots, and adult moths emerge when the rainy season begins. Some *D. lineolata* larvae have asynchronous development and develop slower or faster than the rest of the population; asynchrony would normally be disadvantageous and have negative fitness consequences (van Asch & Visser, 2007; Mopper, 2005; Singer & Parmesan, 2010). For example, adult *D. lineolata* which emerge as corn plants senesce would oviposit on corn but larvae would not survive. The availability of a novel host plant such as sorghum when corn was no longer available would provide a potential host plant for *D. lineolata* oviposition, larval feeding and reproduction. The corn and sorghum populations of *D. lineolata* from La Union are separated in time as the two host plants grow in the rainy and dry season. Availability of a novel host plant such as sorghum at the start of the dry season could impose strong selection on moths to adapt to available host plant resources. Host plant shifts can occur in sympatry. For example, the pea aphid, *Acyrthosiphon pisum* Harris which was introduced into the USA about 100 years ago, shows host plant preferences for alfalfa and red clover when there is no spatial separation of the two host plant species (Via, 1999).

The mechanisms that may contribute to host‐associated differentiation and maintenance of the isolation of insects on two or more host plant have been explored in other systems. Insects feeding on a novel host plants can develop host fidelity with oviposition preference for the natal plant (Diehl & Bush 1984; Wood et al., 1999), and assortative mating could occur between individuals from the same host plant. Host plant fidelity could be tested for *D. lineolata* populations from corn and sorghum, to determine whether adult moths prefer to oviposit on or prefer the odor of their respective host plants. *Rhagoletis pomonella* host plant‐associated populations prefer the odors of their natal host plant and avoid odors of non‐natal host plants (Forbes, Fisher, & Feder, 2005). Hybrids of two *Rhagoletis* host plant‐associated populations did not prefer the host plant of either parent species; the authors suggest this could lead to inability to find suitable oviposition sites and failure to reproduce, contributing to further isolation of host plant‐associated populations (Linn et al., 2004). McBride and Singer (2010) similarly found that hybrids of host plant‐associated populations of *Euphydryas editha* had intermediate behavioral preferences, leading to reduced performance and reproductive isolation of parental populations. Other behavioral factors that contribute to the isolation of populations are whether or not they have distinct pheromone blends which contribute to assortative mating (Landolt & Phillips, 1997). For example, *Ostrinia nubilalis* has two host plant races in Europe with different pheromone blends, and there is a host plant preference for oviposition on the natal host as well as assortative mating of the two moth types (Bethenod et al., 2005). Pheromones were not investigated for the host plant‐associated populations of *Diatraea lineolata* or *D. saccharalis* in this study, but could be similarly investigated in future studies.

The second moth species, *D. saccharalis*, was found to have two genetically divergent groups through the use of both mitochondrial DNA and AFLP markers. Sugarcane, the primary host plant of *D. saccharalis*, is a perennial and grown year round through the wet and dry seasons. Sugarcane harvest occurs in the beginning of the dry season, but some plant portions along with roots remain in the soil where insects can diapause. Perennials have been suggested to favor insect specialization (Via, 1999). More genetic variability was found in the mitochondrial DNA sequences from *D. saccharalis* individuals than among those of *D. lineolata*. A recent study found evidence for several possible *D. saccharalis* species, but host plant‐associated strains have not been previously investigated (Joyce et al., 2014). *D. saccharalis* mitochondrial DNA sequences from El Salvador had two groups (a large and small clade) that were 1% divergent from each other (Figure 6, Table S1). Most of the sequences from El Salvador (20 of 23 individuals) comprised one large clade, which grouped with individuals previously sequenced from Mexico and the southern United States. The two clades with individuals from El Salvador are not specific to one particular host plant. The El Salvador *D. saccharalis* in the large clade were collected from sites (1–6) from rice, sugarcane, and sorghum. The smaller clade with individuals from El Salvador contained two individuals from sugarcane site 2, and one moth from site 3 on sorghum; no individuals in the small clade were associated with rice. Both clades with *D. saccharalis* individuals from El Salvador contained individuals of the two AFLP genotypes (Figures 5 and 6).

The AFLPs used in the Structure analysis found that all *D. saccharalis* from rice were one genotype, most larvae from sugarcane were one genotype, and collections from sorghum consisted of two genotypes (Figure 5). The AMOVA of the four host plant × site *D. saccharalis* populations found the sugarcane, and sorghum populations were significantly distant from each other, but this was not attributable to isolation by distance. The highest *F*_ST_ values were between sugarcane and the two sorghum populations at San Andres and Santo Tomas (*F*_ST_ 0.133, *F*_ST_ 0.104) (Table 5), followed by an *F*_ST_ of 0.095 between the two sorghum populations (site 3, 4). The AMOVA found that season accounted for 4% of genetic variation among populations (Table 4). The genetic divergence among populations of *D. saccharalis* from sugarcane and sorghum suggests some level of host‐associated differentiation. Host plant phenology may contribute to host‐associated differentiation of *D. saccharalis* populations on sugarcane and sorghum as was suggested for *D. lineolata* on corn and sorghum. The availability of sorghum as a novel host plant at the time of sugarcane harvest provides a host plant resource for *D. saccharalis* oviposition and development, similar to that discussed for *D. lineolata*.

Both *Diatraea* species had lower parasitism rates on sorghum than on the other dominant host plant for each moth (*D. lineolata* corn, *D. saccharalis* sugarcane). Sorghum could provide these *Diatraea* spp. a refuge from parasitism and therefore a fitness advantage for offspring. A previous study of *Diatraea* larvae on corn in El Salvador suggested that parasitoids may be synchronized with their host insects and similarly become dormant in the dry season (Quezada, 1978). Parasitoids in other systems are attracted to the odors associated with their host insects or odors associated with their host herbivores feeding on plants (Vet & Dicke, 1992). It is possible that the parasitoid flies (Tachinidae) attacking *Diatraea* spp. in El Salvador do not yet recognize volatiles emitted from sorghum because it is a novel plant, as this is the cases for some predators that have shifted host plants (Raffa, Powell, & Townsend, 2013).

Both *D. lineolata* and *D. saccharalis* have colonized novel crop plants, which have been introduced for cultivation. *D. lineolata* and *D. saccharalis* both appear to be experiencing some degree of host‐associated differentiation on sorghum, perhaps due to the differences in host plant phenology associated with the corn–sorghum and sugarcane–sorghum cropping cycles between the rainy and dry seasons. The low percent parasitism of each moth species on sorghum suggests that natural enemies have not yet followed their host herbivores onto this relatively new host plant resource. Further experiments could explore the preference and performance of host plant‐associated populations of these two *Diatraea* species, to uncover potential mechanisms, which might contribute to genetic divergence of host plant‐associated populations.

## Conflict of Interest

None declared.

## Data Accessibility

All mitochondrial DNA‐COI sequences have been submitted to GenBank to request accession numbers.

Genbank numbers of *D. saccharalis* in this study include the following: El_Salvador_7.9 KX976522, El_Salvador_7.23 KX976523, El_Salvador_10.6 KX976524, El_Salvador_11.5 KX976525, El_Salvador_11.18 KX976526, El_Salvador_11.47 KX976527, El_Salvador_13.22 KX976528, El_Salvador_13.23 KX976529, El_Salvador_13.25 KX976530, El_Salvador_13.30 KX976531, El_Salvador_13.46 KX976532, El_Salvador_14.5 KX976533, El_Salvador_14.12 KX976534, El_Salvador_16.7 KX976535, El_Salvador_17.76 KX976536, El_Salvador_18.5 KX976537, El_Salvador_19.9 KX976538, El_Salvador_19.14 KX976539, El_Salvador_19.31 KX976540, El_Salvador_20.2 KX976541, El_Salvador_20.9 KX976542, El_Salvador_23.3 KX976543, El_Salvador_24.1 KX976544

Genbank numbers of *D. lineolata* in this study include the following: DL_1.5 KX976545, DL_5.5 KX976546, DL_6.14 KX976547, DL_9.21 KX976548, DL_9.8 KX976549, DL_9.12 KX976550, DL_13.8 KX976551, DL_15.24 KX976552, DL_15.36 KX976553, DL_15.79 KX976554, DL_15.111 KX976555, DL_15.12 KX976556, DL_15.80 KX976557, DL_17.4 KX976558, DL_17.10 KX976559, DL_17.12 KX976560, DL_17.27 KX976561, DL_17.31 KX976562, DL_17.70 KX976563, DL_17.24 KX976564, DL_17.26 KX976565, DL_17.58 KX976566, DL_19.9 KX976567, DL_19.21 KX976568, DL_19.36 KX976569
