# Supplementary material for: Host‐plant associated genetic divergence of two Diatraea spp. (Lepidoptera: Crambidae) stemborers on novel crop plants
Source: Ecol Evol. 2016 Nov 13;6(23):8632–44. doi: 10.1002/ece3.2541 (PMC5167014; doi:10.1002/ece3.2541)
Supplement: Supplementary file 1 [file ECE3-6-8632-s001.docx]

Table S1. The pairwise genetic distance for *D. saccharalis* individuals collected in El Salvador and for others from Genbank or BOLD included in Figure 6. Complete Genbank accession numbers or Bar Code of Life numbers are listed on Figure 6. El Salvador individuals are listed with their original collection codes which are also listed in Figure 6.

|  | | **USFla1** | | **USFla2** | | **Arg008** | | **Arg077** | | **Arg091** | | **ES7.9** | | **ES7.23** | | **ES11.5** | | **ES11.47** | | **ES13.22** | | **ES13.23** | | **ES13.30** | | **ES13.46** | | **ES14.5** | | **ES14.12** | | **ES16.7** | |  | |
| --- | --- | --- | --- | --- | --- | --- | --- | --- | --- | --- | --- | --- | --- | --- | --- | --- | --- | --- | --- | --- | --- | --- | --- | --- | --- | --- | --- | --- | --- | --- | --- | --- | --- | --- | --- |
| **USFLA1** |  | |  | |  | |  | |  | |  | |  | |  | |  | |  | |  | |  | |  | |  | |  | |  | |  | |  |
| **USFLA2** | 0.00 | |  | |  | |  | |  | |  | |  | |  | |  | |  | |  | |  | |  | |  | |  | |  | |  | |  |
| **Arg008** | 0.02 | | 0.03 | |  | |  | |  | |  | |  | |  | |  | |  | |  | |  | |  | |  | |  | |  | |  | |  |
| **Arg077** | 0.02 | | 0.03 | | 0.00 | |  | |  | |  | |  | |  | |  | |  | |  | |  | |  | |  | |  | |  | |  | |  |
| **Arg091** | 0.02 | | 0.03 | | 0.00 | | 0.00 | |  | |  | |  | |  | |  | |  | |  | |  | |  | |  | |  | |  | |  | |  |
| **ES7.9** | 0.02 | | 0.02 | | 0.01 | | 0.02 | | 0.01 | |  | |  | |  | |  | |  | |  | |  | |  | |  | |  | |  | |  | |  |
| **ES7.23** | 0.02 | | 0.02 | | 0.01 | | 0.02 | | 0.01 | | 0.00 | |  | |  | |  | |  | |  | |  | |  | |  | |  | |  | |  | |  |
| **ES11.5** | 0.02 | | 0.02 | | 0.02 | | 0.02 | | 0.02 | | 0.00 | | 0.00 | |  | |  | |  | |  | |  | |  | |  | |  | |  | |  | |  |
| **ES11.47** | 0.02 | | 0.02 | | 0.01 | | 0.02 | | 0.01 | | 0.00 | | 0.00 | | 0.00 | |  | |  | |  | |  | |  | |  | |  | |  | |  | |  |
| **ES13.22** | 0.02 | | 0.02 | | 0.01 | | 0.02 | | 0.01 | | 0.00 | | 0.00 | | 0.00 | | 0.00 | |  | |  | |  | |  | |  | |  | |  | |  | |  |
| **ES13.23** | 0.02 | | 0.02 | | 0.01 | | 0.02 | | 0.01 | | 0.00 | | 0.00 | | 0.00 | | 0.00 | | 0.00 | |  | |  | |  | |  | |  | |  | |  | |  |
| **ES13.30** | 0.02 | | 0.02 | | 0.01 | | 0.02 | | 0.01 | | 0.00 | | 0.00 | | 0.00 | | 0.00 | | 0.00 | | 0.00 | |  | |  | |  | |  | |  | |  | |  |
| **ES13.46** | 0.02 | | 0.02 | | 0.01 | | 0.02 | | 0.01 | | 0.00 | | 0.00 | | 0.00 | | 0.00 | | 0.00 | | 0.00 | | 0.00 | |  | |  | |  | |  | |  | |  |
| **ES14.5** | 0.02 | | 0.02 | | 0.01 | | 0.02 | | 0.01 | | 0.00 | | 0.00 | | 0.00 | | 0.00 | | 0.00 | | 0.00 | | 0.00 | | 0.00 | |  | |  | |  | |  | |  |
| **ES14.12** | 0.02 | | 0.02 | | 0.01 | | 0.02 | | 0.01 | | 0.00 | | 0.00 | | 0.00 | | 0.00 | | 0.00 | | 0.00 | | 0.00 | | 0.00 | | 0.00 | |  | |  | |  | |  |
| **ES16.7** | 0.02 | | 0.02 | | 0.01 | | 0.02 | | 0.01 | | 0.00 | | 0.00 | | 0.00 | | 0.00 | | 0.00 | | 0.00 | | 0.00 | | 0.00 | | 0.00 | | 0.00 | |  | |  | |  |
| **ES17.76** | 0.02 | | 0.02 | | 0.01 | | 0.02 | | 0.01 | | 0.00 | | 0.00 | | 0.00 | | 0.00 | | 0.00 | | 0.00 | | 0.00 | | 0.00 | | 0.00 | | 0.00 | | 0.00 | |  | |  |
| **ES18.5** | 0.02 | | 0.02 | | 0.01 | | 0.02 | | 0.01 | | 0.00 | | 0.00 | | 0.00 | | 0.00 | | 0.00 | | 0.00 | | 0.00 | | 0.00 | | 0.00 | | 0.00 | | 0.00 | |  | |  |
| **ES19.9** | 0.02 | | 0.02 | | 0.01 | | 0.02 | | 0.01 | | 0.00 | | 0.00 | | 0.00 | | 0.00 | | 0.00 | | 0.00 | | 0.00 | | 0.00 | | 0.00 | | 0.00 | | 0.00 | |  | |  |
| **ES19.14** | 0.02 | | 0.02 | | 0.01 | | 0.02 | | 0.01 | | 0.00 | | 0.00 | | 0.00 | | 0.00 | | 0.00 | | 0.00 | | 0.00 | | 0.00 | | 0.00 | | 0.00 | | 0.00 | |  | |  |
| **ES19.31** | 0.02 | | 0.02 | | 0.01 | | 0.02 | | 0.01 | | 0.00 | | 0.00 | | 0.00 | | 0.00 | | 0.00 | | 0.00 | | 0.00 | | 0.00 | | 0.00 | | 0.00 | | 0.00 | |  | |  |
| **ES20.2** | 0.02 | | 0.02 | | 0.01 | | 0.02 | | 0.01 | | 0.00 | | 0.00 | | 0.00 | | 0.00 | | 0.00 | | 0.00 | | 0.00 | | 0.00 | | 0.00 | | 0.00 | | 0.00 | |  | |  |
| **ES20.9** | 0.02 | | 0.02 | | 0.01 | | 0.02 | | 0.01 | | 0.00 | | 0.00 | | 0.00 | | 0.00 | | 0.00 | | 0.00 | | 0.00 | | 0.00 | | 0.00 | | 0.00 | | 0.00 | |  | |  |
| **ES23.3** | 0.02 | | 0.02 | | 0.01 | | 0.02 | | 0.01 | | 0.00 | | 0.00 | | 0.00 | | 0.00 | | 0.00 | | 0.00 | | 0.00 | | 0.00 | | 0.00 | | 0.00 | | 0.00 | |  | |  |
| **ES24.1** | 0.02 | | 0.02 | | 0.01 | | 0.02 | | 0.01 | | 0.00 | | 0.00 | | 0.00 | | 0.00 | | 0.00 | | 0.00 | | 0.00 | | 0.00 | | 0.00 | | 0.00 | | 0.00 | |  | |  |
| **Mex54** | 0.02 | | 0.02 | | 0.01 | | 0.02 | | 0.01 | | 0.00 | | 0.00 | | 0.00 | | 0.00 | | 0.00 | | 0.00 | | 0.00 | | 0.00 | | 0.00 | | 0.00 | | 0.00 | |  | |  |
| **Mex55** | 0.02 | | 0.02 | | 0.01 | | 0.02 | | 0.01 | | 0.00 | | 0.00 | | 0.00 | | 0.00 | | 0.00 | | 0.00 | | 0.00 | | 0.00 | | 0.00 | | 0.00 | | 0.00 | |  | |  |
| **Mex56** | 0.02 | | 0.02 | | 0.01 | | 0.02 | | 0.01 | | 0.00 | | 0.00 | | 0.00 | | 0.00 | | 0.00 | | 0.00 | | 0.00 | | 0.00 | | 0.00 | | 0.00 | | 0.00 | |  | |  |
| **Mex57** | 0.02 | | 0.02 | | 0.01 | | 0.02 | | 0.01 | | 0.00 | | 0.00 | | 0.00 | | 0.00 | | 0.00 | | 0.00 | | 0.00 | | 0.00 | | 0.00 | | 0.00 | | 0.00 | |  | |  |
| **Mex58** | 0.02 | | 0.02 | | 0.01 | | 0.02 | | 0.01 | | 0.00 | | 0.00 | | 0.00 | | 0.00 | | 0.00 | | 0.00 | | 0.00 | | 0.00 | | 0.00 | | 0.00 | | 0.00 | |  | |  |
| **Mex59** | 0.02 | | 0.02 | | 0.01 | | 0.02 | | 0.01 | | 0.00 | | 0.00 | | 0.00 | | 0.00 | | 0.00 | | 0.00 | | 0.00 | | 0.00 | | 0.00 | | 0.00 | | 0.00 | |  | |  |
| **Mex60** | 0.02 | | 0.02 | | 0.01 | | 0.02 | | 0.01 | | 0.00 | | 0.00 | | 0.00 | | 0.00 | | 0.00 | | 0.00 | | 0.00 | | 0.00 | | 0.00 | | 0.00 | | 0.00 | |  | |  |

Table S1, cont.

|  | | **ES17.76** | | **ES18.5** | | **ES19.9** | | **ES19.14** | | **ES19.31** | | **ES20.2** | | **ES20.9** | | **ES23.3** | | **ES24.1** | | **Mex54** | | **Mex55** | | **Mex56** | | **Mex57** | | **Mex58** | | **Mex59** | |  | |
| --- | --- | --- | --- | --- | --- | --- | --- | --- | --- | --- | --- | --- | --- | --- | --- | --- | --- | --- | --- | --- | --- | --- | --- | --- | --- | --- | --- | --- | --- | --- | --- | --- | --- |
| **USFLA1** |  | |  | |  | |  | |  | |  | |  | |  | |  | |  | |  | |  | |  | |  | |  | |  | |  |
| **USFLA2** |  | |  | |  | |  | |  | |  | |  | |  | |  | |  | |  | |  | |  | |  | |  | |  | |  |
| **Arg008** |  | |  | |  | |  | |  | |  | |  | |  | |  | |  | |  | |  | |  | |  | |  | |  | |  |
| **Arg077** |  | |  | |  | |  | |  | |  | |  | |  | |  | |  | |  | |  | |  | |  | |  | |  | |  |
| **Arg091** |  | |  | |  | |  | |  | |  | |  | |  | |  | |  | |  | |  | |  | |  | |  | |  | |  |
| **ES7.9** |  | |  | |  | |  | |  | |  | |  | |  | |  | |  | |  | |  | |  | |  | |  | |  | |  |
| **ES7.23** |  | |  | |  | |  | |  | |  | |  | |  | |  | |  | |  | |  | |  | |  | |  | |  | |  |
| **ES11.5** |  | |  | |  | |  | |  | |  | |  | |  | |  | |  | |  | |  | |  | |  | |  | |  | |  |
| **ES11.47** |  | |  | |  | |  | |  | |  | |  | |  | |  | |  | |  | |  | |  | |  | |  | |  | |  |
| **ES13.22** |  | |  | |  | |  | |  | |  | |  | |  | |  | |  | |  | |  | |  | |  | |  | |  | |  |
| **ES13.23** |  | |  | |  | |  | |  | |  | |  | |  | |  | |  | |  | |  | |  | |  | |  | |  | |  |
| **ES13.30** |  | |  | |  | |  | |  | |  | |  | |  | |  | |  | |  | |  | |  | |  | |  | |  | |  |
| **ES13.46** |  | |  | |  | |  | |  | |  | |  | |  | |  | |  | |  | |  | |  | |  | |  | |  | |  |
| **ES14.5** |  | |  | |  | |  | |  | |  | |  | |  | |  | |  | |  | |  | |  | |  | |  | |  | |  |
| **ES14.12** |  | |  | |  | |  | |  | |  | |  | |  | |  | |  | |  | |  | |  | |  | |  | |  | |  |
| **ES16.7** |  | |  | |  | |  | |  | |  | |  | |  | |  | |  | |  | |  | |  | |  | |  | |  | |  |
| **ES17.76** |  | |  | |  | |  | |  | |  | |  | |  | |  | |  | |  | |  | |  | |  | |  | |  | |  |
| **ES18.5** | 0.00 | |  | |  | |  | |  | |  | |  | |  | |  | |  | |  | |  | |  | |  | |  | |  | |  |
| **ES19.9** | 0.00 | | 0.00 | |  | |  | |  | |  | |  | |  | |  | |  | |  | |  | |  | |  | |  | |  | |  |
| **ES19.14** | 0.00 | | 0.00 | | 0.00 | |  | |  | |  | |  | |  | |  | |  | |  | |  | |  | |  | |  | |  | |  |
| **ES19.31** | 0.00 | | 0.00 | | 0.00 | | 0.00 | |  | |  | |  | |  | |  | |  | |  | |  | |  | |  | |  | |  | |  |
| **ES20.2** | 0.00 | | 0.00 | | 0.00 | | 0.00 | | 0.00 | |  | |  | |  | |  | |  | |  | |  | |  | |  | |  | |  | |  |
| **ES20.9** | 0.00 | | 0.00 | | 0.00 | | 0.00 | | 0.00 | | 0.00 | |  | |  | |  | |  | |  | |  | |  | |  | |  | |  | |  |
| **ES23.3** | 0.00 | | 0.00 | | 0.00 | | 0.00 | | 0.00 | | 0.00 | | 0.00 | |  | |  | |  | |  | |  | |  | |  | |  | |  | |  |
| **ES24.1** | 0.00 | | 0.00 | | 0.00 | | 0.00 | | 0.00 | | 0.00 | | 0.00 | | 0.00 | |  | |  | |  | |  | |  | |  | |  | |  | |  |
| **Mex54** | 0.00 | | 0.00 | | 0.00 | | 0.00 | | 0.00 | | 0.00 | | 0.00 | | 0.00 | | 0.00 | |  | |  | |  | |  | |  | |  | |  | |  |
| **Mex55** | 0.00 | | 0.00 | | 0.00 | | 0.00 | | 0.00 | | 0.00 | | 0.00 | | 0.00 | | 0.00 | | 0.00 | |  | |  | |  | |  | |  | |  | |  |
| **Mex56** | 0.00 | | 0.00 | | 0.00 | | 0.00 | | 0.00 | | 0.00 | | 0.00 | | 0.00 | | 0.00 | | 0.00 | | 0.00 | |  | |  | |  | |  | |  | |  |
| **Mex57** | 0.00 | | 0.00 | | 0.00 | | 0.00 | | 0.00 | | 0.00 | | 0.00 | | 0.00 | | 0.00 | | 0.00 | | 0.00 | | 0.00 | |  | |  | |  | |  | |  |
| **Mex58** | 0.00 | | 0.00 | | 0.00 | | 0.00 | | 0.00 | | 0.00 | | 0.00 | | 0.00 | | 0.00 | | 0.00 | | 0.00 | | 0.00 | | 0.00 | |  | |  | |  | |  |
| **Mex59** | 0.00 | | 0.00 | | 0.00 | | 0.00 | | 0.00 | | 0.00 | | 0.00 | | 0.00 | | 0.00 | | 0.00 | | 0.00 | | 0.00 | | 0.00 | | 0.00 | |  | |  | |  |
| **Mex60** | 0.00 | | 0.00 | | 0.00 | | 0.00 | | 0.00 | | 0.00 | | 0.00 | | 0.00 | | 0.00 | | 0.00 | | 0.00 | | 0.00 | | 0.00 | | 0.00 | | 0.00 | |  | |  |

Table S1, cont.

|  | | **USFla1** | | **USFla2** | | **Arg008** | | **Arg077** | | **Arg091** | | **ES7.9** | | **ES7.23** | | **ES11.5** | | **ES11.47** | | **ES13.22** | | **ES13.23** | | **ES13.30** | | **ES13.46** | | **ES14.5** | | **ES14.12** | | **ES16.7** | |
| --- | --- | --- | --- | --- | --- | --- | --- | --- | --- | --- | --- | --- | --- | --- | --- | --- | --- | --- | --- | --- | --- | --- | --- | --- | --- | --- | --- | --- | --- | --- | --- | --- | --- |
| **Tx166** | 0.02 | | 0.02 | | 0.01 | | 0.02 | | 0.01 | | 0.00 | | 0.00 | | 0.00 | | 0.00 | | 0.00 | | 0.00 | | 0.00 | | 0.00 | | 0.00 | | 0.00 | | 0.00 | |  |
| **Tx1560** | 0.02 | | 0.02 | | 0.01 | | 0.02 | | 0.01 | | 0.00 | | 0.00 | | 0.00 | | 0.00 | | 0.00 | | 0.00 | | 0.00 | | 0.00 | | 0.00 | | 0.00 | | 0.00 | |  |
| **Tx1565** | 0.02 | | 0.02 | | 0.01 | | 0.02 | | 0.01 | | 0.00 | | 0.00 | | 0.00 | | 0.00 | | 0.00 | | 0.00 | | 0.00 | | 0.00 | | 0.00 | | 0.00 | | 0.00 | |  |
| **USLA2** | 0.02 | | 0.02 | | 0.01 | | 0.02 | | 0.01 | | 0.00 | | 0.00 | | 0.00 | | 0.00 | | 0.00 | | 0.00 | | 0.00 | | 0.00 | | 0.00 | | 0.00 | | 0.00 | |  |
| **Bol260** | 0.03 | | 0.03 | | 0.01 | | 0.01 | | 0.01 | | 0.02 | | 0.02 | | 0.02 | | 0.02 | | 0.02 | | 0.02 | | 0.02 | | 0.02 | | 0.02 | | 0.02 | | 0.02 | |  |
| **Bol275** | 0.03 | | 0.03 | | 0.01 | | 0.01 | | 0.01 | | 0.02 | | 0.02 | | 0.02 | | 0.02 | | 0.02 | | 0.02 | | 0.02 | | 0.02 | | 0.02 | | 0.02 | | 0.02 | |  |
| **Braz76** | 0.03 | | 0.03 | | 0.00 | | 0.01 | | 0.00 | | 0.02 | | 0.02 | | 0.02 | | 0.02 | | 0.02 | | 0.02 | | 0.02 | | 0.02 | | 0.02 | | 0.02 | | 0.02 | |  |
| **Braz77** | 0.03 | | 0.03 | | 0.01 | | 0.01 | | 0.01 | | 0.02 | | 0.02 | | 0.02 | | 0.02 | | 0.02 | | 0.02 | | 0.02 | | 0.02 | | 0.02 | | 0.02 | | 0.02 | |  |
| **Braz78** | 0.03 | | 0.03 | | 0.00 | | 0.01 | | 0.00 | | 0.02 | | 0.02 | | 0.02 | | 0.02 | | 0.02 | | 0.02 | | 0.02 | | 0.02 | | 0.02 | | 0.02 | | 0.02 | |  |
| **Braz79** | 0.03 | | 0.03 | | 0.00 | | 0.01 | | 0.00 | | 0.02 | | 0.02 | | 0.02 | | 0.02 | | 0.02 | | 0.02 | | 0.02 | | 0.02 | | 0.02 | | 0.02 | | 0.02 | |  |
| **Braz80** | 0.03 | | 0.03 | | 0.00 | | 0.01 | | 0.00 | | 0.02 | | 0.02 | | 0.02 | | 0.02 | | 0.02 | | 0.02 | | 0.02 | | 0.02 | | 0.02 | | 0.02 | | 0.02 | |  |
| **Braz81** | 0.03 | | 0.03 | | 0.00 | | 0.01 | | 0.00 | | 0.02 | | 0.02 | | 0.02 | | 0.02 | | 0.02 | | 0.02 | | 0.02 | | 0.02 | | 0.02 | | 0.02 | | 0.02 | |  |
| **Braz82** | 0.03 | | 0.03 | | 0.00 | | 0.01 | | 0.00 | | 0.02 | | 0.02 | | 0.02 | | 0.02 | | 0.02 | | 0.02 | | 0.02 | | 0.02 | | 0.02 | | 0.02 | | 0.02 | |  |
| **Braz83** | 0.03 | | 0.03 | | 0.00 | | 0.01 | | 0.00 | | 0.02 | | 0.02 | | 0.02 | | 0.02 | | 0.02 | | 0.02 | | 0.02 | | 0.02 | | 0.02 | | 0.02 | | 0.02 | |  |
| **Braz84** | 0.03 | | 0.03 | | 0.00 | | 0.01 | | 0.00 | | 0.02 | | 0.02 | | 0.02 | | 0.02 | | 0.02 | | 0.02 | | 0.02 | | 0.02 | | 0.02 | | 0.02 | | 0.02 | |  |
| **Braz85** | 0.03 | | 0.03 | | 0.01 | | 0.01 | | 0.01 | | 0.02 | | 0.02 | | 0.02 | | 0.02 | | 0.02 | | 0.02 | | 0.02 | | 0.02 | | 0.02 | | 0.02 | | 0.02 | |  |
| **Braz86** | 0.03 | | 0.03 | | 0.00 | | 0.01 | | 0.00 | | 0.02 | | 0.02 | | 0.02 | | 0.02 | | 0.02 | | 0.02 | | 0.02 | | 0.02 | | 0.02 | | 0.02 | | 0.02 | |  |
| **USTxE1** | 0.03 | | 0.03 | | 0.03 | | 0.03 | | 0.02 | | 0.01 | | 0.01 | | 0.01 | | 0.01 | | 0.01 | | 0.01 | | 0.01 | | 0.01 | | 0.01 | | 0.01 | | 0.01 | |  |
| **USTxE2** | 0.03 | | 0.03 | | 0.03 | | 0.03 | | 0.02 | | 0.01 | | 0.01 | | 0.01 | | 0.01 | | 0.01 | | 0.01 | | 0.01 | | 0.01 | | 0.01 | | 0.01 | | 0.01 | |  |
| **USTxS1** | 0.03 | | 0.03 | | 0.03 | | 0.03 | | 0.03 | | 0.01 | | 0.01 | | 0.01 | | 0.01 | | 0.01 | | 0.01 | | 0.01 | | 0.01 | | 0.01 | | 0.01 | | 0.01 | |  |
| **USTxS2** | 0.03 | | 0.03 | | 0.03 | | 0.03 | | 0.03 | | 0.01 | | 0.01 | | 0.01 | | 0.01 | | 0.01 | | 0.01 | | 0.01 | | 0.01 | | 0.01 | | 0.01 | | 0.01 | |  |
| **ES10.6** | 0.03 | | 0.03 | | 0.02 | | 0.02 | | 0.02 | | 0.01 | | 0.01 | | 0.01 | | 0.01 | | 0.01 | | 0.01 | | 0.01 | | 0.01 | | 0.01 | | 0.01 | | 0.01 | |  |
| **ES11.18** | 0.03 | | 0.03 | | 0.02 | | 0.02 | | 0.02 | | 0.01 | | 0.01 | | 0.01 | | 0.01 | | 0.01 | | 0.01 | | 0.01 | | 0.01 | | 0.01 | | 0.01 | | 0.01 | |  |
| **ES13.25** | 0.03 | | 0.03 | | 0.02 | | 0.02 | | 0.02 | | 0.01 | | 0.01 | | 0.01 | | 0.01 | | 0.01 | | 0.01 | | 0.01 | | 0.01 | | 0.01 | | 0.01 | | 0.01 | |  |
| **USLA1** | 0.03 | | 0.03 | | 0.02 | | 0.02 | | 0.02 | | 0.01 | | 0.01 | | 0.01 | | 0.01 | | 0.01 | | 0.01 | | 0.01 | | 0.01 | | 0.01 | | 0.01 | | 0.01 | |  |

Table S1, cont.

|  | | **ES17.76** | | **ES18.5** | | **ES19.9** | | **ES19.14** | | **ES19.31** | | **ES20.2** | | **ES20.9** | | **ES23.3** | | **ES24.1** | | **Mex54** | | **Mex55** | | **Mex56** | | **Mex57** | | **Mex58** | | **Mex59** | | **Mex60** | |
| --- | --- | --- | --- | --- | --- | --- | --- | --- | --- | --- | --- | --- | --- | --- | --- | --- | --- | --- | --- | --- | --- | --- | --- | --- | --- | --- | --- | --- | --- | --- | --- | --- | --- |
| **Tx166** | 0.00 | | 0.00 | | 0.00 | | 0.00 | | 0.00 | | 0.00 | | 0.00 | | 0.00 | | 0.00 | | 0.00 | | 0.00 | | 0.00 | | 0.00 | | 0.00 | | 0.00 | | 0.00 | |  |
| **Tx1560** | 0.00 | | 0.00 | | 0.00 | | 0.00 | | 0.00 | | 0.00 | | 0.00 | | 0.00 | | 0.00 | | 0.00 | | 0.00 | | 0.00 | | 0.00 | | 0.00 | | 0.00 | | 0.00 | |  |
| **Tx1565** | 0.00 | | 0.00 | | 0.00 | | 0.00 | | 0.00 | | 0.00 | | 0.00 | | 0.00 | | 0.00 | | 0.00 | | 0.00 | | 0.00 | | 0.00 | | 0.00 | | 0.00 | | 0.00 | |  |
| **USLA2** | 0.00 | | 0.00 | | 0.00 | | 0.00 | | 0.00 | | 0.00 | | 0.00 | | 0.00 | | 0.00 | | 0.00 | | 0.00 | | 0.00 | | 0.00 | | 0.00 | | 0.00 | | 0.00 | |  |
| **Bol260** | 0.02 | | 0.02 | | 0.02 | | 0.02 | | 0.02 | | 0.02 | | 0.02 | | 0.02 | | 0.02 | | 0.02 | | 0.02 | | 0.02 | | 0.02 | | 0.02 | | 0.02 | | 0.02 | |  |
| **Bol275** | 0.02 | | 0.02 | | 0.02 | | 0.02 | | 0.02 | | 0.02 | | 0.02 | | 0.02 | | 0.02 | | 0.02 | | 0.02 | | 0.02 | | 0.02 | | 0.02 | | 0.02 | | 0.02 | |  |
| **Braz76** | 0.02 | | 0.02 | | 0.02 | | 0.02 | | 0.02 | | 0.02 | | 0.02 | | 0.02 | | 0.02 | | 0.02 | | 0.02 | | 0.02 | | 0.02 | | 0.02 | | 0.02 | | 0.02 | |  |
| **Braz77** | 0.02 | | 0.02 | | 0.02 | | 0.02 | | 0.02 | | 0.02 | | 0.02 | | 0.02 | | 0.02 | | 0.02 | | 0.02 | | 0.02 | | 0.02 | | 0.02 | | 0.02 | | 0.02 | |  |
| **Braz78** | 0.02 | | 0.02 | | 0.02 | | 0.02 | | 0.02 | | 0.02 | | 0.02 | | 0.02 | | 0.02 | | 0.02 | | 0.02 | | 0.02 | | 0.02 | | 0.02 | | 0.02 | | 0.02 | |  |
| **Braz79** | 0.02 | | 0.02 | | 0.02 | | 0.02 | | 0.02 | | 0.02 | | 0.02 | | 0.02 | | 0.02 | | 0.02 | | 0.02 | | 0.02 | | 0.02 | | 0.02 | | 0.02 | | 0.02 | |  |
| **Braz80** | 0.02 | | 0.02 | | 0.02 | | 0.02 | | 0.02 | | 0.02 | | 0.02 | | 0.02 | | 0.02 | | 0.02 | | 0.02 | | 0.02 | | 0.02 | | 0.02 | | 0.02 | | 0.02 | |  |
| **Braz81** | 0.02 | | 0.02 | | 0.02 | | 0.02 | | 0.02 | | 0.02 | | 0.02 | | 0.02 | | 0.02 | | 0.02 | | 0.02 | | 0.02 | | 0.02 | | 0.02 | | 0.02 | | 0.02 | |  |
| **Braz82** | 0.02 | | 0.02 | | 0.02 | | 0.02 | | 0.02 | | 0.02 | | 0.02 | | 0.02 | | 0.02 | | 0.02 | | 0.02 | | 0.02 | | 0.02 | | 0.02 | | 0.02 | | 0.02 | |  |
| **Braz83** | 0.02 | | 0.02 | | 0.02 | | 0.02 | | 0.02 | | 0.02 | | 0.02 | | 0.02 | | 0.02 | | 0.02 | | 0.02 | | 0.02 | | 0.02 | | 0.02 | | 0.02 | | 0.02 | |  |
| **Braz84** | 0.02 | | 0.02 | | 0.02 | | 0.02 | | 0.02 | | 0.02 | | 0.02 | | 0.02 | | 0.02 | | 0.02 | | 0.02 | | 0.02 | | 0.02 | | 0.02 | | 0.02 | | 0.02 | |  |
| **Braz85** | 0.02 | | 0.02 | | 0.02 | | 0.02 | | 0.02 | | 0.02 | | 0.02 | | 0.02 | | 0.02 | | 0.02 | | 0.02 | | 0.02 | | 0.02 | | 0.02 | | 0.02 | | 0.02 | |  |
| **Braz86** | 0.02 | | 0.02 | | 0.02 | | 0.02 | | 0.02 | | 0.02 | | 0.02 | | 0.02 | | 0.02 | | 0.02 | | 0.02 | | 0.02 | | 0.02 | | 0.02 | | 0.02 | | 0.02 | |  |
| **USTxE1** | 0.01 | | 0.01 | | 0.01 | | 0.01 | | 0.01 | | 0.01 | | 0.01 | | 0.01 | | 0.01 | | 0.01 | | 0.01 | | 0.01 | | 0.01 | | 0.01 | | 0.01 | | 0.01 | |  |
| **USTxE2** | 0.01 | | 0.01 | | 0.01 | | 0.01 | | 0.01 | | 0.01 | | 0.01 | | 0.01 | | 0.01 | | 0.01 | | 0.01 | | 0.01 | | 0.01 | | 0.01 | | 0.01 | | 0.01 | |  |
| **USTxS1** | 0.01 | | 0.01 | | 0.01 | | 0.01 | | 0.01 | | 0.01 | | 0.01 | | 0.01 | | 0.01 | | 0.01 | | 0.01 | | 0.01 | | 0.01 | | 0.01 | | 0.01 | | 0.01 | |  |
| **USTxS2** | 0.01 | | 0.01 | | 0.01 | | 0.01 | | 0.01 | | 0.01 | | 0.01 | | 0.01 | | 0.01 | | 0.01 | | 0.01 | | 0.01 | | 0.01 | | 0.01 | | 0.01 | | 0.01 | |  |
| **ES10.6** | 0.01 | | 0.01 | | 0.01 | | 0.01 | | 0.01 | | 0.01 | | 0.01 | | 0.01 | | 0.01 | | 0.01 | | 0.01 | | 0.01 | | 0.01 | | 0.01 | | 0.01 | | 0.01 | |  |
| **ES11.18** | 0.01 | | 0.01 | | 0.01 | | 0.01 | | 0.01 | | 0.01 | | 0.01 | | 0.01 | | 0.01 | | 0.01 | | 0.01 | | 0.01 | | 0.01 | | 0.01 | | 0.01 | | 0.01 | |  |
| **ES13.25** | 0.01 | | 0.01 | | 0.01 | | 0.01 | | 0.01 | | 0.01 | | 0.01 | | 0.01 | | 0.01 | | 0.01 | | 0.01 | | 0.01 | | 0.01 | | 0.01 | | 0.01 | | 0.01 | |  |
| **USLA1** | 0.01 | | 0.01 | | 0.01 | | 0.01 | | 0.01 | | 0.01 | | 0.01 | | 0.01 | | 0.01 | | 0.01 | | 0.01 | | 0.01 | | 0.01 | | 0.01 | | 0.01 | | 0.01 | |  |

Table S1, cont.

|  | | **Tx166** | | **Tx1560** | | **Tx1565** | | **USLA2** | | **Bol260** | | **Bol275** | | **Braz76** | | **Braz77** | | **Braz78** | | **Braz79** | | **Braz80** | | **Braz81** | | **Braz82** | | **Braz83** | | **Braz84** | | **Braz85** | |
| --- | --- | --- | --- | --- | --- | --- | --- | --- | --- | --- | --- | --- | --- | --- | --- | --- | --- | --- | --- | --- | --- | --- | --- | --- | --- | --- | --- | --- | --- | --- | --- | --- | --- |
| **Tx166** |  | |  | |  | |  | |  | |  | |  | |  | |  | |  | |  | |  | |  | |  | |  | |  | |  |
| **Tx1560** | 0.00 | |  | |  | |  | |  | |  | |  | |  | |  | |  | |  | |  | |  | |  | |  | |  | |  |
| **Tx1565** | 0.00 | | 0.00 | |  | |  | |  | |  | |  | |  | |  | |  | |  | |  | |  | |  | |  | |  | |  |
| **USLA2** | 0.00 | | 0.00 | | 0.00 | |  | |  | |  | |  | |  | |  | |  | |  | |  | |  | |  | |  | |  | |  |
| **Bol260** | 0.02 | | 0.02 | | 0.02 | | 0.02 | |  | |  | |  | |  | |  | |  | |  | |  | |  | |  | |  | |  | |  |
| **Bol275** | 0.02 | | 0.02 | | 0.02 | | 0.02 | | 0.00 | |  | |  | |  | |  | |  | |  | |  | |  | |  | |  | |  | |  |
| **Braz76** | 0.02 | | 0.02 | | 0.02 | | 0.02 | | 0.01 | | 0.01 | |  | |  | |  | |  | |  | |  | |  | |  | |  | |  | |  |
| **Braz77** | 0.02 | | 0.02 | | 0.02 | | 0.02 | | 0.01 | | 0.01 | | 0.00 | |  | |  | |  | |  | |  | |  | |  | |  | |  | |  |
| **Braz78** | 0.02 | | 0.02 | | 0.02 | | 0.02 | | 0.01 | | 0.01 | | 0.00 | | 0.00 | |  | |  | |  | |  | |  | |  | |  | |  | |  |
| **Braz79** | 0.02 | | 0.02 | | 0.02 | | 0.02 | | 0.01 | | 0.01 | | 0.00 | | 0.00 | | 0.00 | |  | |  | |  | |  | |  | |  | |  | |  |
| **Braz80** | 0.02 | | 0.02 | | 0.02 | | 0.02 | | 0.01 | | 0.01 | | 0.00 | | 0.00 | | 0.00 | | 0.00 | |  | |  | |  | |  | |  | |  | |  |
| **Braz81** | 0.02 | | 0.02 | | 0.02 | | 0.02 | | 0.01 | | 0.01 | | 0.00 | | 0.00 | | 0.00 | | 0.00 | | 0.00 | |  | |  | |  | |  | |  | |  |
| **Braz82** | 0.02 | | 0.02 | | 0.02 | | 0.02 | | 0.01 | | 0.01 | | 0.00 | | 0.00 | | 0.00 | | 0.00 | | 0.00 | | 0.00 | |  | |  | |  | |  | |  |
| **Braz83** | 0.02 | | 0.02 | | 0.02 | | 0.02 | | 0.01 | | 0.01 | | 0.00 | | 0.00 | | 0.00 | | 0.00 | | 0.00 | | 0.00 | | 0.00 | |  | |  | |  | |  |
| **Braz84** | 0.02 | | 0.02 | | 0.02 | | 0.02 | | 0.01 | | 0.01 | | 0.00 | | 0.00 | | 0.00 | | 0.00 | | 0.00 | | 0.00 | | 0.00 | | 0.00 | |  | |  | |  |
| **Braz85** | 0.02 | | 0.02 | | 0.02 | | 0.02 | | 0.01 | | 0.01 | | 0.00 | | 0.00 | | 0.00 | | 0.00 | | 0.00 | | 0.00 | | 0.00 | | 0.00 | | 0.00 | |  | |  |
| **Braz86** | 0.02 | | 0.02 | | 0.02 | | 0.02 | | 0.01 | | 0.01 | | 0.00 | | 0.00 | | 0.00 | | 0.00 | | 0.00 | | 0.00 | | 0.00 | | 0.00 | | 0.00 | | 0.00 | |  |
| **USTxE1** | 0.01 | | 0.01 | | 0.01 | | 0.01 | | 0.03 | | 0.03 | | 0.03 | | 0.03 | | 0.03 | | 0.03 | | 0.03 | | 0.03 | | 0.03 | | 0.03 | | 0.03 | | 0.03 | |  |
| **USTxE2** | 0.01 | | 0.01 | | 0.01 | | 0.01 | | 0.03 | | 0.03 | | 0.03 | | 0.03 | | 0.03 | | 0.03 | | 0.03 | | 0.03 | | 0.03 | | 0.03 | | 0.03 | | 0.03 | |  |
| **USTxS1** | 0.01 | | 0.01 | | 0.01 | | 0.01 | | 0.03 | | 0.03 | | 0.03 | | 0.03 | | 0.03 | | 0.03 | | 0.03 | | 0.03 | | 0.03 | | 0.03 | | 0.03 | | 0.03 | |  |
| **USTxS2** | 0.01 | | 0.01 | | 0.01 | | 0.01 | | 0.03 | | 0.03 | | 0.03 | | 0.03 | | 0.03 | | 0.03 | | 0.03 | | 0.03 | | 0.03 | | 0.03 | | 0.03 | | 0.03 | |  |
| **ES10.6** | 0.01 | | 0.01 | | 0.01 | | 0.01 | | 0.02 | | 0.02 | | 0.02 | | 0.02 | | 0.02 | | 0.02 | | 0.02 | | 0.02 | | 0.02 | | 0.02 | | 0.02 | | 0.02 | |  |
| **ES11.18** | 0.01 | | 0.01 | | 0.01 | | 0.01 | | 0.02 | | 0.02 | | 0.02 | | 0.02 | | 0.02 | | 0.02 | | 0.02 | | 0.02 | | 0.02 | | 0.02 | | 0.02 | | 0.02 | |  |
| **ES13.25** | 0.01 | | 0.01 | | 0.01 | | 0.01 | | 0.02 | | 0.02 | | 0.02 | | 0.02 | | 0.02 | | 0.02 | | 0.02 | | 0.02 | | 0.02 | | 0.02 | | 0.02 | | 0.02 | |  |
| **USLA1** | 0.01 | | 0.01 | | 0.01 | | 0.01 | | 0.02 | | 0.02 | | 0.02 | | 0.02 | | 0.02 | | 0.02 | | 0.02 | | 0.02 | | 0.02 | | 0.02 | | 0.02 | | 0.02 | |  |

Table S1, cont.

|  | | **Braz86** | | **USTxE1** | | **USTxE2** | | **USTxS1** | | **USTxS2** | | **ES10.6** | | **ES11.18** | | **ES13.25** | |
| --- | --- | --- | --- | --- | --- | --- | --- | --- | --- | --- | --- | --- | --- | --- | --- | --- | --- |
| **Tx166** |  | |  | |  | |  | |  | |  | |  | |  | |  |
| **Tx1560** |  | |  | |  | |  | |  | |  | |  | |  | |  |
| **Tx1565** |  | |  | |  | |  | |  | |  | |  | |  | |  |
| **USLA2** |  | |  | |  | |  | |  | |  | |  | |  | |  |
| **Bol260** |  | |  | |  | |  | |  | |  | |  | |  | |  |
| **Bol275** |  | |  | |  | |  | |  | |  | |  | |  | |  |
| **Braz76** |  | |  | |  | |  | |  | |  | |  | |  | |  |
| **Braz77** |  | |  | |  | |  | |  | |  | |  | |  | |  |
| **Braz78** |  | |  | |  | |  | |  | |  | |  | |  | |  |
| **Braz79** |  | |  | |  | |  | |  | |  | |  | |  | |  |
| **Braz80** |  | |  | |  | |  | |  | |  | |  | |  | |  |
| **Braz81** |  | |  | |  | |  | |  | |  | |  | |  | |  |
| **Braz82** |  | |  | |  | |  | |  | |  | |  | |  | |  |
| **Braz83** |  | |  | |  | |  | |  | |  | |  | |  | |  |
| **Braz84** |  | |  | |  | |  | |  | |  | |  | |  | |  |
| **Braz85** |  | |  | |  | |  | |  | |  | |  | |  | |  |
| **Braz86** |  | |  | |  | |  | |  | |  | |  | |  | |  |
| **USTxE1** | 0.03 | |  | |  | |  | |  | |  | |  | |  | |  |
| **USTxE2** | 0.03 | | 0.00 | |  | |  | |  | |  | |  | |  | |  |
| **USTxS1** | 0.03 | | 0.00 | | 0.00 | |  | |  | |  | |  | |  | |  |
| **USTxS2** | 0.03 | | 0.00 | | 0.00 | | 0.00 | |  | |  | |  | |  | |  |
| **ES10.6** | 0.02 | | 0.02 | | 0.02 | | 0.02 | | 0.02 | |  | |  | |  | |  |
| **ES11.18** | 0.02 | | 0.02 | | 0.02 | | 0.02 | | 0.02 | | 0.00 | |  | |  | |  |
| **ES13.25** | 0.02 | | 0.02 | | 0.02 | | 0.02 | | 0.02 | | 0.00 | | 0.00 | |  | |  |
| **USLA1** | 0.02 | | 0.02 | | 0.02 | | 0.02 | | 0.02 | | 0.00 | | 0.00 | | 0.00 | |  |
